# Whiteboard animation videos for increasing awareness about antimicrobial resistance, prudent antimicrobial prescribing, and urinary tract infection prevention

**DOI:** 10.1017/ash.2023.185

**Published:** 2023-06-15

**Authors:** Vinesh Patel, Sarah Chadwick, Mina Bakhit, Gillian Hawksworth, Mamoon A. Aldeyab

**Affiliations:** 1 Department of Microbiology, Mid-Yorkshire Hospitals NHS Trust, Pinderfields Hospital, Wakefield, United Kingdom; 2 West Yorkshire Health and Care Partnership, Wakefield, United Kingdom; 3 Institute for Evidence-Based Healthcare, Bond University, Gold Coast, Queensland, Australia; 4 Department of Pharmacy, School of Applied Sciences, University of Huddersfield, Huddersfield, United Kingdom

*To the Editor—*Bacterial antimicrobial resistance (AMR) has emerged worldwide as one of the leading public health threats of the 21st century.^1^ A 2016 review on antimicrobial resistance commissioned by the UK government estimated that global AMR deaths could reach 10 million people per year by 2050.^2^ Decreasing unnecessary antimicrobial use reduces the risk of antibiotic resistance but also reduces unnecessary side effects and cost; furthermore it helps maintain a healthy individual human microbiome.^3^ The West Yorkshire Health and Care Partnership (WYHCP) AMR program aims to achieve at least a 10% reduction in antimicrobial resistance infections by 2024.^4^ Behavioral change by the general public is a key part of achieving this goal. The (C), opportunity (O), and motivation (M) as three key factors capable of changing behavior (B) (ie, COM-B) model of behavioral change conceptualizes how capabilities, opportunities and motivations combine to cause behavior.^4^ Using this model, we designed a study to help develop educational videos for the public—specifically, whiteboard animation videos. These animations are a visually engaging way of presenting complex information, including health topics. Whiteboard animations about healthcare have gained hundreds of millions of views, and there is evidence that they have improved patient understanding of a number of topics.^5^

We studied 2 key areas of public behavior: reducing consumption of unnecessary antibiotics and drinking enough fluids daily to help prevent urinary tract infections (UTIs). To inform our study methods, we consulted the Behavioural Science and Insights Unit (UK Health Security Agency). We chose a focus-group workshop study design to produce qualitatively rich data. Convenience sampling was used to recruit a heterogeneous sample of participants for the focus group; initially by approaching students and colleagues within the University of Huddersfield campus, then inviting friends, friends of friends, and family to take part. All interested participants who verbally confirmed that they held no healthcare qualifications were provided or shown written project information, then they were contacted via email with a copy of the project information sheet, workshop details, and a consent form (Appendix 1). Written consent was required to be completed prior to participation and was additionally collected verbally prior to audio-recording their session.

Demographic information was collected from participants via a pseudonymized online questionnaire link, including age, gender, first part of address code, and ethnicity. Using the COM-B model,^4^ we formed a semistructured interview guide (Appendix 2), designed to explore the both the underlying reasons for people’s behavior and how we could change them using animations (Table 1). The questions were developed to ensure the interviewer had various ways to solicit more depth in responses.

**Table 1.** Focus-Group Questions Developed in Accordance With the COM-B Model, and Corresponding Intervention Considerations After Data Analysis

Issue Underlying Behavior	COM-B Questions	Structured Focus Group Question	Intervention Development Need
**Related to reducing consumption of unnecessary antibiotics**			
Lack of awareness of what antibiotics are and what they should be used for	Capability (psychological): Are you clear about the signs of infection? Are you aware of what antibiotics are? Do you know what they can or cannot treat? Opportunity (physical & social): Can you access antibiotics? Is taking antibiotics influenced by peers, relatives’ other social influences)? Motivation (reflective): How do you decide that antibiotics are needed?	Can you tell me a bit about a time you or someone you know had to take antibiotics for an infection? Probe: How long did it last? Did you self-care? Take antibiotics? Seek advice? How should people decide they need antibiotics?	Messaging and imagery should be clear and concrete.
Lack of awareness that antibiotics can cause harm (benefits of avoiding antibiotics)	Capability (psychological): Do you know antibiotics can cause harm? Opportunity (physical): Are there competing priorities? Motivation (reflective): Are antibiotics a concern or issue to you?	What do you think about taking antibiotics? Probe: Do you have any concerns about taking antibiotics?	Messaging should provide information that taking antibiotics will have an impact.
Lack of awareness of when taking antibiotics is appropriate	Capability (psychological): Do you know when antibiotics are appropriate? Opportunity (physical & social): Do you have access to healthcare, access to antibiotics? Is accessing antibiotics influenced by peers, relatives’ other social influences? Motivation (reflective): Why would you ask for antibiotics?	Would you ask your doctor, pharmacist, dentist for antibiotics? Probe: Is there anywhere else would you get antibiotics from? Would you consider using unprescribed antibiotics? Is yes, why?	Messaging should highlight possible losses of not taking action.
Lack of awareness about self-care and antibiotic alternatives	Capability (psychological): How do you decide what action is appropriate? Are you aware of what I need to do? Opportunity (physical): Can you access over the counter products? Motivation (reflective): Do you know what will happen if you try antibiotic alternatives? Will alternative treatments work or make things worse? Is it a priority for you?	If you feel you have an infection, what can you do instead of taking antibiotics? Probe: What sort of things do you do to try to help your symptoms, if at all? Would you drink more liquids? Rest? Take paracetamol or ibuprofen or other pain relief? Over the counter or other products? Where would you get advice about this?	Messaging should highlight possible gains of taking action.
Lack of concern about antibiotic resistance	Capability (psychological): Do you understand what AMR means? Motivation (reflective): Does antibiotic resistance apply to you? Do you have concerns/fears around AMR? Will your actions make a difference to AMR?	What do the words “antibiotic resistance” mean to you? Probe: How could antibiotic resistance affect you or your loved ones? How about the community?	Messaging should highlight that changes in behavior will have an impact on AMR (and will be worthwhile).
Lack of trust in healthcare advice provided	Capability (physical): Can you access health information? Opportunity (physical): Do you have time to look for health information related to AMR? Motivation (reflective & automatic): Is AMR information a priority? Is AMR a key health issue to you? Do you trust health information?	Where would you get information related to AMR from? Probe: The Internet and/or your healthcare provider? Why?	NHS trusted imagery should be used with clear and concrete messaging.
Lack of awareness that AMR is relevant to self, family, community	Motivation (automatic & reflective): Does the animation style feel relatable to you? Is it self-relevant? Will your actions make a difference to AMR? Do you feel motivated by messaging highlighting possible gains of taking action or possible losses of not taking action?	[shown animation stills] What message do you think the drawings are trying to get across? Probe: Do you like the characters? How does each example make you feel? What is each drawing trying to tell you? Is it helpful? Things you like and don’t like about the way the drawing/chart looks; (color, layout, characters, and message). Do you prefer one drawing more than the other and why?	Imagery should reflect community and highlight that behavior will have an impact (and will be worthwhile).
Lack of ability to access health information	Capability (physical): Can you access health information on video/you tube?	Do you foresee any difficulties in using such a video resource? Probe: Follow up on issues raised.	Design should include films shorts for digital social media strategy.
**Related to drinking enough fluids daily to help prevent urinary tract infections (UTIs)**			
Lack of awareness of and confidence using tools to identify signs of dehydration	Capability (psychological): What are tools such as a urine color chart telling you? Motivation (reflective): Would this tool be an incentive to act?	Urine chart: Do you think of this chart? Prompt: What is it trying to tell you? Is it helpful? The things you like about the way the chart looks (color, layout, font); things you don’t you like about the way the chart looks? (color, layout, font)	Messaging should highlight that behavior (using the chart) is easy and achievable Messaging should highlight possible gains of taking action.
Lack of awareness of how keeping hydrated promotes good health	Capability (psychological): Do you know how to stay hydrated? Motivation (autonomic): What would incentivize or stop you drinking enough?	How many nonalcoholic drinks (like water, juice tea, coffee) do you think an adult should drink in one day? Prompt: What would help you to drink the right amount each day? What types of drinks and food do you think help us stay hydrated? What would stop you drinking more water?	Messaging should highlight that keeping hydrated is easy and achievable.

The study included 6 participants of 3 different ethnicities: African, White British, and White and Black Caribbean. Age categories ranged from <18 years to >60 years; 67% identified as male and 33% identified as female. The focus group was audio-recorded, transcribed verbatim, and then analyzed thematically, using Microsoft Excel (Redmond, WA), by independent researchers with expertise in qualitative research (M.B. and M.A.A.).^6^

Our data analysis revealed the following 4 themes: (1) Patients would try alternative treatments (eg, paracetamol, lozenges, or honey) before seeking antibiotics from their primary healthcare provider or getting it from other sources (including leftovers from previous infections). (2) Online health information is used as a triage, with uncertainty about the trustworthiness of online sources (other than NHS, BBC news, and medical journals). (3) There is a lack of knowledge about and fear of antibiotic resistance and its impact on individuals and society. (4) There is uncertainty regarding the required amount of fluid intake and the usefulness of using a urine color chart (Appendix 3). However, participants thought the videos were easy to use and could be used for education. Further, the focus-group participants discussed key behaviors driving change. Scripts were designed to motivate listeners about how infection and AMR will affect themselves and others. The scripts emphasized the potential losses if the behavior is unchanged and highlighted the benefits of changing behavior.

Our study informed the production of 2 videos, one outlining the size of the problem of antimicrobial resistance and the behaviors that can prevent it (https://www.youtube.com/watch?v=XaSCJvYk23s) and another that outlined how to help prevent infections (especially UTIs) by remaining hydrated (https://www.youtube.com/watch?v=ljdQxkJtdvY). To interpret the study, we must consider its limitations. The study could be expanded to a larger number of participants and could be extended to include vulnerable populations (older adults, non–English-language speakers and low-income groups) most at risk from antibiotic overprescribing and health inequities. The focus group took place during the COVID-19 pandemic, and this and other factors may have introduced bias regarding who decided to participate such as people vulnerable to infection concerned about meeting others, or their caregivers. An evaluation of the videos is still required to measure the attributes they have in changing behavior.

Using the COM-B framework,^4^ we identified important AMR behavioral factors to target: lack of knowledge (of what AMR is and how much fluid to drink daily), emotional response (fear of the damage AMR will do in future), and existing behaviors (trying to find reliable information online, trying alternative treatments before antibiotics). Overall, the coherent evidence regarding efficacy and best practice for educational videos targeting antibiotic behaviors remains limited.^7^ Indeed, some AMR evidence points to how public education videos should be integrated with antibiotic policies in healthcare.^8^ It is important to consider how we use the videos (eg, internet upload only or community engagement^9^). Evidence regarding AMR education is increasing, and based on user data, studies such as ours help inform the public regarding health-related activities as we try to mobilize forces effectively to avert a major future health crisis from AMR.^10^

## Supplementary material

For supplementary material accompanying this paper visit http://doi.org/10.1017/ash.2023.185. click here to view supplementary material

## References

[1] Murray CJ , Ikuta KS , Sharara F , et al, Global burden of bacterial antimicrobial resistance in 2019: a systematic analysis. Lancet 2022;399:629–655. 35065702 10.1016/S0140-6736(21)02724-0 PMC8841637

[2] O’Neill J. Tackling drug-resistant infections globally: final report and recommendations. *Review on Antimicrobial Resistance* website. https://amr-review.org/sites/default/files/160518_Final%20paper_with%20cover.pdf. Published 2016. Accessed May 30, 2023.

[3] Patangia DV , Anthony Ryan C , Dempsey E , Paul Ross R , Stanton C. Impact of antibiotics on the human microbiome and consequences for host health. Microbiol Open 2022;11:e1260. 10.1002/mbo3.1260 PMC8756738 35212478

[4] West R , Michie S. A brief introduction to the COM-B Model of behaviour and the PRIME theory of motivation. *Qeios* 2020. doi: 10.32388/WW04E6.

[5] Ciciriello S , Johnston RV , Osborne RH , et al. Multimedia educational interventions for consumers about prescribed and over-the-counter medications. Cochrane Database Syst Rev 2013;4:CD008416. 10.1002/14651858.CD008416.pub2 PMC11222367 23633355

[6] Maguire M , Delahunt B. Doing a thematic analysis: a practical, step-by-step guide for learning and teaching scholars. All Irel J High Educ 2017;9:3.

[7] Parveen S , Garzon-Orjuela N , Amin D , McHugh P , Vellinga A. Public health interventions to improve antimicrobial resistance awareness and behavioural change associated with antimicrobial use: a systematic review exploring the use of social media. Antibiotics (Basel) 2022;11:669. 35625313 10.3390/antibiotics11050669 PMC9137793

[8] Hawkins O , Scott AM , Montgomery A , et al. Comparing public attitudes, knowledge, beliefs and behaviours towards antibiotics and antimicrobial resistance in Australia, United Kingdom, and Sweden (2010–2021): a systematic review, meta-analysis, and comparative policy analysis. PLoS One 2022;17: e0261917. 35030191 10.1371/journal.pone.0261917 PMC8759643

[9] Appiah B , Asamoah-Akuoko L , Samman E , et al. The impact of antimicrobial resistance awareness interventions involving schoolchildren, development of an animation and parents engagements: a pilot study. Antimicrob Resist Infect Control 2022;11:26. 35120562 10.1186/s13756-022-01062-6 PMC8817549

[10] Courtenay M , Castro-Sanchez E , Fitzpatrick M , Gallagher R , Lim R , Morris G. Tackling antimicrobial resistance 2019–2024—The UK’s five-year national action plan. J Hosp Infect 2019;101:426–427. 30826342 10.1016/j.jhin.2019.02.019

