# Supplementary material for: Whiteboard animation videos for increasing awareness about antimicrobial resistance, prudent antimicrobial prescribing, and urinary tract infection prevention
Source: Antimicrob Steward Healthc Epidemiol. 2023 Jun 15;3(1):e101. doi: 10.1017/ash.2023.185 (PMC10311686; doi:10.1017/ash.2023.185)
Supplement: Supplementary file 1 [file S2732494X23001857sup001.docx]

**Appendix 1: Participant information sheet and consent form**

**Who are we and what is the purpose of our project?**

We are the Improving Population Heath team, part of the NHS West Yorkshire Health and Care Partnership (WY HCP) ICS. We are working to improve public health services for our local population. We want to hear from people from our community as it’s essential to making sure our healthcare services produce good content. The purpose of this project is to make some educational animation videos, and the subject of these videos are how we can prevent infections, particularly infections hard to treat with antibiotics.

Our job is to listen to your opinions and suggestions and use them to help make the videos.

**When and where are we asking people to take part in?**

We are running the focus group from on Tuesday 7^th^ June at 5 pm at University of Huddersfield campus.

**Who else will be there?**

Our team at the NHS are collaborating with Pharmacy researchers at the University of Huddersfield that will be present at the focus group.

**What are we asking people to take part in?**

To help us design the videos, we are asking people to take part in a focus group. In the focus group, we will talk about what you know about antibiotic resistance and other ways in which we can prevent infection and ask for your opinions on the way the animation will be designed.

Your name and personal details will not be recorded. At the start of the session, we will ask you to anonymously record some details about your background including age, gender, ethnicity, and first part of your postcode. We are collecting this information so that we know how to use the answers from the focus group to help produce the videos, which will be designed to reach people of all backgrounds.

The session will last 1 hour and will take place at the University of Huddersfield. To thank participants for taking part we are offering compensation to the value of £25 as well as travel expenses.

**Is my feedback confidential?**

Yes, when your focus group recording is written up your name will not be included. No names will be used at any point in our write up, or any reports that are produced. We do however have a duty to report harm, therefore if you mention something to us during your focus group that indicates you, or someone else, is at risk of harm, we will need to share that information with other statutory services.

**Can I change my mind?**

Your involvement in this project is completely voluntary. If you wish to stop the focus group at any point during the focus group, you can. You can also withdraw your consent for your focus group feedback to be used in the project up to two days after the focus group date. You can do this by contacting the WY HCP project lead Vinesh Patel at [vinesh.patel2@nhs.net](mailto:vinesh.patel2@nhs.net) or Dr M. Al Deyab at University of Huddersfield [M.AlDeyab@hud.ac.uk](mailto:M.AlDeyab@hud.ac.uk)

Two days after the focus group, we will start grouping feedback together. At this stage, it will not be possible to withdraw your consent because no names will be used when grouping participant feedback, so we will not be able to identify what things you may have said to remove them.

**How will my feedback be recorded and stored?**

With your permission, we will make an audio recording (NOT visual) of the focus group when it takes place. This is so we can concentrate on our conversations with you, rather than trying to write detailed notes of what you are saying. This recording will be securely shared with a transcription company that specialises in writing up confidential health recordings/discussions, this will ensure we have an accurate record of your feedback. We have a data processing agreement in place with this company to ensure your information is treated in the strictest confidence, and all audio recordings are encrypted when they are shared.

The focus groups notes will be stored securely in line with the Record Management Code of Practice for Health and Social Care and after they are transcribed, they will be confidentially destroyed immediately.

**What will happen to the focus group results?**

We will review and group all the feedback from the participants who take part in the project and write a summary of the findings. At no point will any of our reports from the project include any names of participants. The project findings will be reported back to the project lead at West Yorkshire Health and Care Partnership Trust and other health professionals supporting the reduction in antimicrobial resistance.

**Who do I contact if I have any questions about the project?**

If you would like to discuss taking part in the project, or for any other questions you may have, please contact us at Dr Vinesh Patel on [vinesh.patel2@nhs.net](mailto:vinesh.patel2@nhs.net) or Dr M Al Deyab on [M.AlDeyab@hud.ac.uk](mailto:M.AlDeyab@hud.ac.uk)

**What do I do if I want to make a complaint about the project?**

In the first instance contact the AMR programme manager for an informal discussion on [sarah.chadwick9@nhs.net](mailto:sarah.chadwick9@nhs.net) or contact the WY HCP team on [Westyorkshire.ICS@nhs.net](mailto:Westyorkshire.ICS@nhs.net)

**West Yorkshire Health & Care Partnership Antibiotic Resistance Animation Videos:-**

**Participant Consent Form**

| **Please indicate you agree to the following statements by ticking ‘Yes’:** | Yes | No |
| --- | --- | --- |
| I have read the participant information sheet and I understand the purpose of the project: |  |  |
| I agree to take part in this focus group: |  |  |
| I agree for my contribution to the focus group to be anonymously audio recorded and written up by the transcription company: |  |  |
| I understand my participation is voluntary: |  |  |
| I understand that participant’s names won’t be used in the focus group and that I will be asked not to tell anyone outside the focus group who attended the session and what other participants said |  |  |
| I understand I can withdraw my contribution in the two days following the focus group date, and that after this date my anonymous contribution cannot be withdrawn: |  |  |
| I understand my focus group recording will be securely stored and destroyed in line with the Record Management Code of Practice for Health and Social Care: |  |  |
| I understand my name will not be included in the research analysis and reports: |  |  |

Your privacy is important to us, by signing this form you are consenting to the Project Team to hold and process your personal data as described in the participant information sheet and as confirmed in the table above.

Please note you will additionally be asked to give your verbal consent at the start of the focus group.

| **Participant Name (Print):** |  |
| --- | --- |
| **Participant Signature:** |  |
| **Participant email:** |  |
| **Participant telephone number:** |  |
| **Date:** |  |

| **Session lead Name (Print):** |  |
| --- | --- |
| **Session lead Signature:** |  |
| **Date:** |  |

# **Appendix 2** **Semi-structured guide for focus-group**

**Session Introduction – 10 minutes**

Start of focus group

• Introduce yourself and thank you for your participation e.g.

- Good evening, thank you for coming along today we’re very grateful to you
- My name is Dr Vinesh Patel, and this is Professor Gillian Hawksworth, Sarah Chadwick and Dr Mamoon Al-Deyab. We are from the project teams at West Yorkshire Health and Care Partnership (the local NHS public health group) and University of Huddersfield.

**House keeping**

Before we start there are a few things I want to say:

Our discussion today is due to last around 1 hour, ending about 6.30pm. If you can please stay behind for 10 minutes afterwards we will confirm arrangements for sending you the vouchers and travel compensation.

We are not expecting a fire alarm but if the alarm does sound you should [describe route to nearest fire exit].

There are toilets located [show and describe route to toilets]; if you would like to go at any point in the session, please feel free to let us know, and then return to the session.

The research is totally confidential –Your views will be anonymous; we won’t use any names when we write our report. Please don’t use your names at all this evening or in future regarding this session. We also ask that you don’t tell anyone outside this room about who attended or what other participants said.

During the discussion different views may come up, please don’t interrupt, give each other time to speak; we are interested in hearing all views (there are no right or wrong answers).

If you need to leave before the end of the session, please let Sarah Chadwick know, but do please try to stay as it may disrupt the session and we do really want to hear your views.

**Purpose**

So, just before we start the discussion then, just a reminder about why we are doing this focus group. We want to tackle antibiotic resistance as a high priority in West Yorkshire, by raising awareness about it and letting people know the things they can do to prevent getting infections of various kinds. This focus group will help us understand the awareness and behaviours regarding around antibiotics and infection in West Yorkshire.

**Consent**

Before we start today, we need to make sure everyone has read the information sheet we provided and completed a permission form to say you are all happy to take part, and for the anonymous audio recording of the session. Can I just check those have been completed? (If not, can we please get these completed now?)

Great. We can now start the focus group. I am switching on the recorder now.

- *Vinesh: “For the purposes of this audio recording, all participants of the workshop here today have confirmed they consent to be recorded today”.*

**Focus group questions**

Thank you all for agreeing to participate in this focus group, we’re very grateful for your time.

So, this evening, I’d really like to ask your views on Antibiotic Resistance. I’m going to start off quite general and then discuss some specific areas. There are no right or wrong answers, we’re all just giving our ideas today.

1. So, remembering not to mention any names, can you tell me a bit about a time you or someone you know had to take antibiotics for an infection? *Probe: How long did it last? Did you self-care? Take antibiotics? Seek advice? How should people decide they need antibiotics?*
2. What do you think about taking antibiotics? *Probe: do you have any concerns about taking antibiotics?*
3. Would you ask your doctor, pharmacist, dentist for antibiotics? *Probe: Is there anywhere else would you get antibiotics from? Would you consider using unprescribed antibiotics? Is yes - why?*
4. If you feel you have an infection, what can you do instead of taking antibiotics? *Probe: What sort of things do you do to try to help your symptoms, if at all? Would you drink more liquids? Rest? Take paracetamol / ibuprofen or other pain relief? Over the counter/other products? Where would you get advice about this?*
5. What do the words “antibiotic resistance” mean to you? *Probe: how could antibiotic resistance affect you or your loved ones? How about the community?*
6. Where would you get information related to AMR from? *Probe:* *the internet, your healthcare provider?*

Before we move on, does everyone want to add anything else?

Thanks everyone, I’m going to pause the recording for a 5-minute break

- **BREAK - for 5 minutes**

Ok, I’m just going to restart the recording. Welcome back everyone. So now we’re going to talk about lifestyle and prevention of a very common and important infection – urine infections also known as “UTIs”, urinary tract infections.

One way people can prevent getting a urine infection is by increasing the amount of fluid we drink each day (other than alcohol) – keeping hydrated. Having enough water in our bodies helps our urine system stay healthy and free of infection. One way of measuring our hydration levels is urine colour, as you can see on this chart:

7. So next question is, what do you think of this chart? *Prompt:* *what is it trying to tell you? Is it helpful?* *The things you like about the way the chart looks (colour, layout, font); things you don’t you like about the way the chart looks? (colour, layout, font)*

8. How many non-alcoholic drinks (like water, juice tea, coffee) do you think an adult should drink in one day? Try and get range of views. *Prompt: What would help you to drink the right amount each day?* *What types of drinks and food do you think help us stay hydrated?* *What would stop you drinking more water?*

The reason we wanted to get all these opinions here today is, we’re make some educational material for people to learn about drug resistant infections how we can prevent them. The animation is currently being developed. We would like your thoughts on these drawings which may or may not be used.

Here, hand out sheet to show the participants the animation stills below – ask participant to think aloud their thoughts:


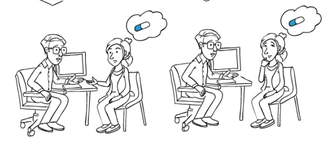
9a. Image 1: What message do you think the drawings are trying to get across? *Probe: Do you like the characters? How does each example make you feel? What is each drawing trying to tell you? Is it helpful? The things you like about the way the drawing looks (colour, layout, characters, message); things you don’t you like about the way the chart looks? (colour, layout, characters, message)*


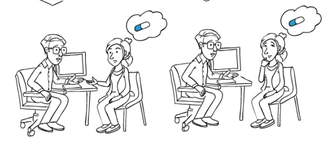
9b. Image 2: What message do you think the drawings are trying to get across? *Probe: Do you like the characters? How does each example make you feel? What is each drawing trying to tell you? Is it helpful? The things you like about the way the drawing looks (colour, layout, characters, message); things you don’t you like about the way the chart looks? (colour, layout, characters, message)*

9c. Do you prefer one drawing more than the other? Please let us know A or B and Why.

10. Do you foresee any difficulties in using such a video resource? *Probe: follow up on issues raised.*

11. Do you have any other thoughts or comments that we haven’t touched upon?

**Thank you for your time, the recording will now be stopped**

**Appendix 3** Figure with screenshot from Hydration videos - urine colour comparison frame showing dehydration


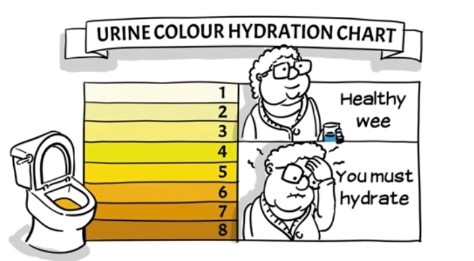


Figure 1. Screenshot from Hydration videos - urine colour chart comparison frame showing dehydration
